# Fast Personal Protective Equipment Detection for Real Construction Sites Using Deep Learning Approaches

**DOI:** 10.3390/s21103478

**Published:** 2021-05-17

**Authors:** Zijian Wang, Yimin Wu, Lichao Yang, Arjun Thirunavukarasu, Colin Evison, Yifan Zhao

**Affiliations:** 1 School of Civil Engineering, Central South University, Changsha 410075, China; zijianwang@csu.edu.cn (Z.W.); wuyimin531@163.com (Y.W.); 2 School of Aerospace, Transport and Manufacturing, Cranfield University, Bedfordshire MK43 0AL, UK; lichao.yang@cranfield.ac.uk; 3 BAM Nuttall, St James House, Knoll Road, Camberley GU15 3XW, UK; Arjun.Thirunavukarasu@bamnuttall.co.uk (A.T.); colin.Evison@bam.com (C.E.)

**Keywords:** PPE, construction safety, deep learning, You Only Look Once (YOLO), image dataset, real-time detection

## Abstract

The existing deep learning-based Personal Protective Equipment (PPE) detectors can only detect limited types of PPE and their performance needs to be improved, particularly for their deployment on real construction sites. This paper introduces an approach to train and evaluate eight deep learning detectors, for real application purposes, based on You Only Look Once (YOLO) architectures for six classes, including helmets with four colours, person, and vest. Meanwhile, a dedicated high-quality dataset, CHV, consisting of 1330 images, is constructed by considering real construction site background, different gestures, varied angles and distances, and multi PPE classes. The comparison result among the eight models shows that YOLO v5x has the best mAP (86.55%), and YOLO v5s has the fastest speed (52 FPS) on GPU. The detection accuracy of helmet classes on blurred faces decreases by 7%, while there is no effect on other person and vest classes. And the proposed detectors trained on the CHV dataset have a superior performance compared to other deep learning approaches on the same datasets. The novel multiclass CHV dataset is open for public use.

## 1. Introduction

**Figure 1:**
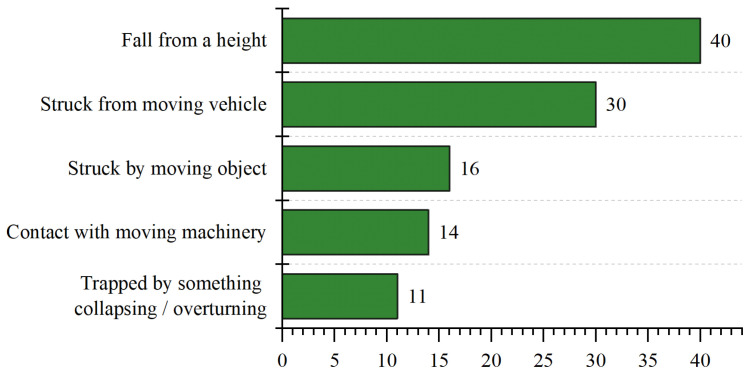
Main kinds of fatal accident for workers in 2018/19 in the UK [3].

Construction usually involves high-risk activities requiring workers to operate at dangerous places and be exposed to risk. Based on the United States’ Bureau of Labor Statistics, the fatalities number increases gradually from 985 in 2015 to 1038 in 2018, with an increase of 2% every year [1]. In China, 840 workers died during construction activities in 2018, and 52.2% of them were caused by falling from a high place [2]. Similarly, according to the UK Health and Safety Executive (HSE), 147 workers suffered from fatal injuries in the UK in 2018/2019, where falling from a high place is the most significant kind of fatal accident [3], as shown in Figure 1. However, the majority of injuries, illness and fatalities could be avoided if workers wear suitable PPE, duch as helmets, safety glasses, gloves, and so on [4]. The helmet is an essential piece of PPE, which protects construction workers by resisting objects and absorbing shock from direct blows to the head by objects. Previous research shows that wearing helmets is an effective way to reduce the probability of skull fracture, neck sprain, and concussion when falling from a height place [5]. Meanwhile, helmets could also reduce the likelihood of severe brain injury by up to 95% for concrete block impacts [6].

**Figure 2:**
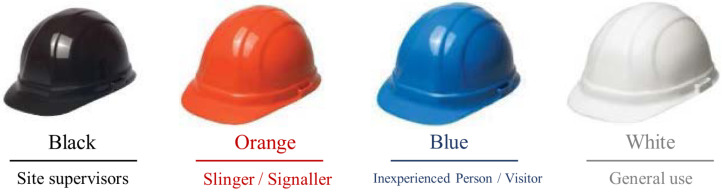
Helmet colors and the roles on construction sites [7].

The main goal of PPE detection is to measure health and safety compliance to improve construction safety. Wearing helmets would reduce the injuries and even fatalities when meeting accidents. Meanwhile, another necessary PPE, the vest, is also required to be worn on construction sites for increasing visibility. The vest with flash lines would help others locate construction workers and avoid accidents, particularly in poor weather, like rainy and foggy days. Another aim is to understand the activities of works and optimize management. The helmet colors present different roles in different countries. Taking the UK as an example, site supervisors usually wear black helmets. Slingers and signallers could wear an orange one. Inexperienced persons or visitors should wear blue helmets. The white helmet is typically for general use, including the manager, client, competent operative and, as shown in Figure 2. Monitoring the helmet and its color is helpful to analyze the activity between different roles, and thus useful to optimize the construction procedures and improve management efficiency and productivity.

The existing PPE detection techniques could be categorized into sensor-based and vision-based methods. The **sensor-based methods** usually adopt positioning technology to track workers and PPE. Zhang et al. [8] used the Global Position System (GPS) to locate workers and helmets. Meanwhile, Kelm et al. [9] designed a mobile Radio Frequency Identification (RFID) portal for checking the PPE compliance. When workers wearing RFID PPE pass through checking gates, the PPE information can be recorded. Furthermore, Zhang et al. [10] combined the RFID technology with the Internet of Things (IoT), where all data would be uploaded to the cloud and shared through web and mobile applications. However, this approach requires workers to wear an extra device for sending and receiving data. Sensor-based helmet detection methods rely on equipment, which are not affected by external factors like weather, illumination, humidity, etc. Therefore, they usually achieve a stable performance and can be applied to most construction sites. However, sensor-based approaches require a large and long-term investment in purchasing, installing, and maintaining. Although a single sensor’s price is relatively low, installing this for every PPE and every worker still requires a large budget, which suggests a limited scalability. Besides this, workers need to wear an end device for connection with the network in current RFID approaches [10], which increases the weight and causeans inconvenience to workers.

The **vision-based methods** adopt cameras to collect images of construction sites, then process them for PPE detection. Images provide rich information that can be utilized to understand complex construction sites more promptly, precisely and comprehensively [11]. Some researchers focus on 3D images. For instance, Han et al. [12] set fixed stereo cameras, JVC 3D Everio camcorder, to record videos of workers in laboratory experiments. Then, actions were reconstructed from videos for safety analysis. However, this can only capture short-range views due to the limitations of stereo cameras. Similarly, a laser scanner was adopted by Cheng et al. [13] to conduct a real-time safety check of workers. Instead of 3D images, computer vision techniques were adopted to detect helmets from 2D images in another research field. Zhu et al. [14] applied histograms of oriented gradient (HOG) to extract head features, which were then fed into a Support Vector Machine (SVM) to classify whether one person wears a helmet or not. Similarly, Rubaiyat et al. [15] combined HOG and SVM to detect human beings, then Circle Hough Transform (CHT) was utilized to detect helmets. Besides, Shrestha et al. [16] implemented edge detection for the head, face, and helmet. Instead of recognizing shapes, Du et al. [17] presented a detection system based on colors. They set color thresholds for different objects, including the face and helmet. The system could output the detection results according to the color value. Many researchers have applied the deep learning techniques into PPE detection to achieve an automated and efficient monitoring process. Nath et al. [18] matched an unknown input PPE image with a previous known image to search the possible PPE information (type and color) in the input image. Wu et al. [19] adopted K-Nearest Neighbors (KNN) to capture moving objects from videos, which were then input into CNN models for classification of the pedestrian, head, and helmet. Similarly, Pradana et al. [20] used a CNN-based model to classify twelve situations, which were the combination of five PPE, such as glasses and helmet. However, the experiments only tested the images with a pure-color indoor background (not real construction sites), which might limit further deployment in outdoor environment. Meanwhile, Akbarzadeh et al. [21] adopted two Faster R-CNN models to detect safety noncompliances, where the first model detects human bodies on the construction site, and the second one detects the helmet and vest. Wu et al. [22] adopted Single Shot MultiBox Detector (SSD) to detect whether construction personnel wear helmets and the corresponding colors. Chen et al. [23] detected the gestures to determine whether workers wore the helmet properly. In 2018, Xie et al. [24] compared the performance of different detection approaches based on the same datasets. You Only Look Once (YOLO) has the best mean average precision (53.8%) and the fastest speed (10 FPS) compared with SSD and faster R-CNN.

Many studies adopt sensor-based or vision-based approaches to achieve real-time and accurate PPE detection, while there are still some gaps. (1) Current PPE transfer learning research can detect limited types of PPE. Most of them are designed for helmet detection, not applicable for other general protective equipment, like the vest, gloves and glasses. Meanwhile, most of them cannot detect the PPE colors. (2) Although there are several studies which apply deep learning detectors to PPE detection, there is still room for improving their performance. (3) There is no PPE detection particularly on blurring face images. With the development of privacy protection, more and more applications require the sensitive facial information to be hidden. To the best of our knowledge, there is no research reported on this topic to date. (4) There are limited high-quality open PPE datasets. Although there are several published PPE researcher works, there are a few open public PPE datasets. Additionally, some of the images in these open datasets are used for advertisements, where the model stands with a standard gesture in front of the camera and the background is not a real construction site. Meanwhile, there is no open dataset which labels different PPE classes and helmet colours. The previous dataset construction did not consider including many different types of image, with different backgrounds, gestures, angles and distances, and multiple classes.

Based on the knowledge gaps discussed above, a high-quality dataset which considers the construction site background, different gestures, angles and so on, is formed in this paper. Additionally, we report a high-efficiency PPE detector, which can predict multiple PPE classes including helmet color with a better performance in terms of correctness and speed by comparing this with the state-of-the-art. Meanwhile, the model would be tested on blurring face images.

## 2. Methodology

### 2.1. Framework

**Figure 3:**
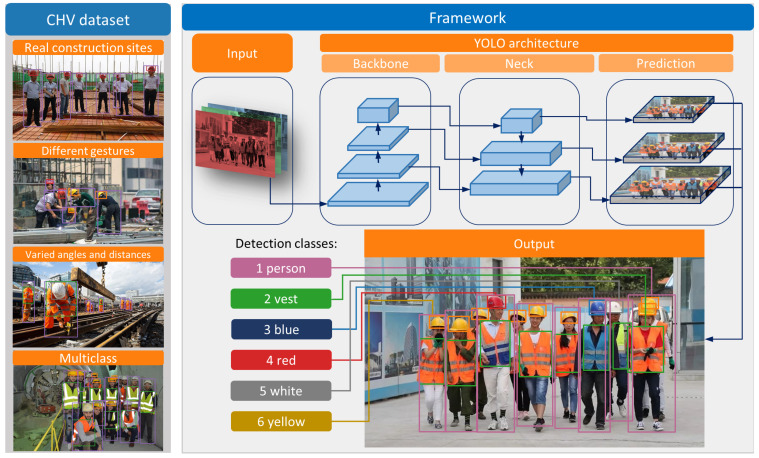
The proposed framework of PPE detection based on YOLO models.

YOLO is adopted as the primary model for PPE detection in this paper, the framework of which is shown in Figure 3. Although different versions of YOLO models have slightly varied architectures, all of them contain three main parts, including backbone, neck, and prediction, in Figure 3. After feeding one image into the YOLO models, the output could be any combination of six desired classes (person, vest, blue helmet, red helmet, white helmet, and yellow helmet). Additionally, a novel dataset, Color Helmet and Vest (CHV), is constructed to detect the helmet, helmet colors, and the vest, where the dataset images are selected from ten thousand images based on four aspects to ensure the model’s robustness.

### 2.2. YOLO Family

#### 2.2.1. YOLO v3 and Previous Versions

YOLO is a powerful real-time object detection system, first presented by Redmon in 2016 [25]. Then, he adjusted the structures to detect more classes with a faster speed, and presented YOLO 9000 [26] in 2017 and YOLO v3 [27] in 2018. Unlike the two-stage models containing searching and classifying regions, YOLO only owns a one-stage process. It applies a single neural network to the full image, where the network divides the image into regions and predicts bounding boxes and probabilities for each region. Meanwhile, YOLO makes predictions with a single network evaluation, unlike other systems, such as R-CNN, which requires thousands for a single image. The unique structure makes YOLO v3 faster than other models (e.g., more than 1000 times faster than R-CNN and 100 times faster than Fast R-CNN [27]).

#### 2.2.2. YOLO v4 and YOLO v5

In 2020, Bochkovskiy et al. [28] released a new version, YOLO v4, which improves the average precision (AP) and FPS by 10% and 12% respectively, compared to YOLO v3 [28]. The main difference between YOLO v3 and YOLO v4 is the adjusted network structure and increased number of applied tricks. For the structure, YOLO v4 changed the backbone from the previous Darknet53 to the current CSPDarknet53. Meanwhile, a few data augmentation tricks are adopted, including Random Erase, Cutout, Hide and Seek, Grid Mask, MixUp, Cutmix, Self-Adversarial Training, Class label smoothing, and Mosaic data augmentation.

**Figure 4:**
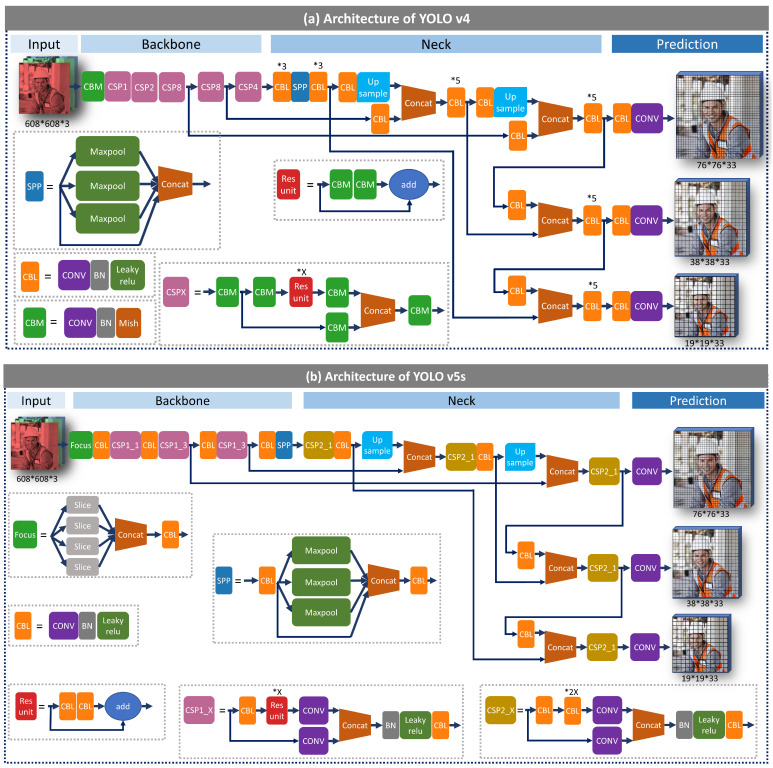
Architectures of YOLO v4 and YOLO v5s.

More recently, a company, Ultralytics, carried out YOLO v5 [29] soon after the release of YOLO v4. Instead of publishing YOLO v5-related work as a research paper, the company only released YOLO v5’s source code at Github. The implementation of YOLO v5 is further explained below, based on the source code. The architectures of YOLO v4 and YOLO v5s are presented in Figure 4. First, only leaky relu activation function (CBL module) is adopted in the hidden layers in YOLO v5, while YOLO v4 has two modules with leaky relu and mish activation functions (CBL and CBM). Second, YOLO v5 adopts a new module at the beginning of the backbone, Focus, which slices input images into four small ones and concatenates them together for convolution operation. For instance, a 608 × 608 × 3 image is split into four small images with the size of 304 × 304 × 3, which then are concatenated into a 304 × 304 × 12 image. Third, YOLO v5 designs two CSPNet modules for the backbone and neck, respectively. CSPNet is presented by Wang et al. [30] to integrate feature maps from the beginning and the end of a network stage to reduce the computation and maintain the processing accuracy. The designed CSPNet module (CSP2_x in Figure 4b), in the YOLO v5 neck stage, strengthens the network feature fusion compared with the normal convolution module in YOLO v4. Except for the structure adjustments, YOLO v5 adopts an algorithm for automatically learning bounding box anchors in the input stage, which could calculate the anchor box’s size for different image sizes and contribute to the detection quality. Besides this, YOLO v5 selects GIoU loss [30] (Equation (1)) as the bounding box regression loss function instead of CIoU loss in YOLO v4 (Equation (2)). GIoU [30] is carried out to solve the inaccurate calculation of non-overlapping bounding boxes that existed in the previous IoU loss function. Furthermore, CIoU considers three geometric factors, including the overlapping area, distance and aspect ratio. CIoU enhances the speed and accuracy to better distinguish difficult regression cases. Lastly, YOLO v5 is constructed under a new training environment at Pytorch [31], making the training processing more friendly than at Darknet.

(1) $$L_{GIoU}=1-IoU+\frac{|C-B\cup B^{gt}|}{\left|C\right|}$$

where $B^{gt}$ is the ground-truth box, *B* is the predicted box, $IoU=\frac{B\cap B^{gt}}{B\cup B^{gt}}$, and *C* is the smallest box covering *B* and $B^{gt}$.

(2) $$L_{CIoU}=1-IoU+\frac{\rho^{2}\left(p,p^{gt}\right)}{c^{2}}+\alpha V$$

where *p* and $p^{gt}$ are the central points of boxes *B* and $B^{gt}$, *c* is the diagonal length of *C*, ρ is specified as Euclidean distance, α and *V* is the consistency of aspect ratio.

### 2.3. CHV Dataset

A novel dataset is constructed to detect the vest, helmet colors and person for this project, named the CHV dataset. Strict criteria are applied to ensure the model’s robustness during real applications. The considered factors include (1) related construction background, (2) people’s gestures, (3) object angles and distances, and (4) number of classes. For some existing datasets [22,32], most images were collected from advertisement, where the background of some images is not construction sites. The CHV dataset would like to collect real construction site images. Meanwhile, the dataset contains different gestures (e.g., bending, kneeling), varied angles (e.g., left, right, front, back, top) and distances (e.g., close range, far away distance). Besides this, during collection, an image containing many classes is preferred. Specifically, the definition of vest in CHV is that top clothes with horizontal and (or) vertical flash lines, rather than the traditional division of the sleeve length. All images in the CHV dataset are collected from online public datasets, GDUT-HWD and SHWD [33]. Based on the discussed criteria, 1330 images which meet the requirements are selected from around 10,000.

**Figure 5:**
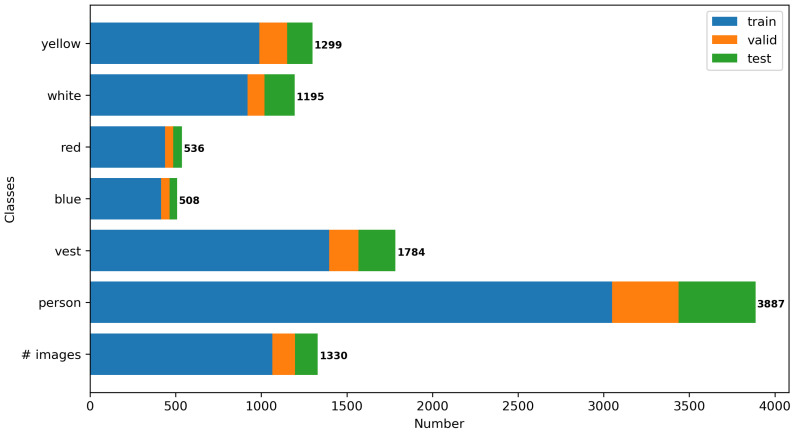
Number of images and instances in the training, validation and test datasets.

After collecting the images, the next step is to annotate them manually using the graphical image annotation tool LabelImg [34]. The annotation classes include 0-person, 1-vest, 2-blue, 3-red, 4-white, 5-yellow. To achieve the best annotation results, another researcher validated the labelled dataset after the initial annotation. CHV dataset contain 1330 images, and 9209 instances in total, where 80% of them (1064 images) were selected randomly as the training dataset, 10% (133 images) as validation, and the remaining 10% (133 images) as the test dataset. The test dataset was only used to evaluate the model performance after training, as shown in Figure 5.

**Figure 6:**
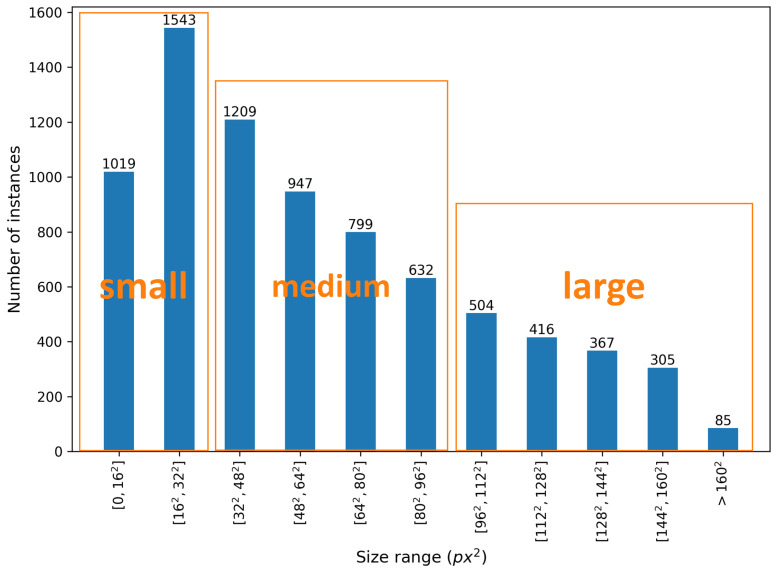
Number of instances in each scale category (calculated on resizing each image to 408 × 408).

The size distribution of instances is also calculated and presented in Figure 6, where the thresholds to divide them into three groups are suggested by Wu et al. [22]. Notably, the small-scale category (area $\leq 32^{2}$$px^{2}$) occupies a large part of the CHV dataset, which adds challenges for the detector but is more appropriate for images from CCTV cameras with a long working distance. There are 3587 medium-scale instances ($32^{2}$$px^{2}$≤area $\leq 96^{2}$$px^{2}$), and 1677 large-scale instances (area $\geq 96^{2}$$px^{2}$).

### 2.4. PPE Detector

#### 2.4.1. Experiment Goals

This paper contains three parts for testing different goals, as shown in Table 1. Specifically, the first goal is to evaluate the performance of different layers on YOLO v3 models. The default YOLO v3 model has three detection layers (13 × 13, 26 × 26, 52 × 52). In theory, a lager detection layer can recognize more object details and improve the detection performance for small objects. Based on this idea, another two layers, 104 × 104 and 208 × 208, are added to the default YOLO v3 (3 layers) model. Therefore, YOLO v3 (five layers) have five prediction layers on five scales from 13 × 13 to 208 × 208. Although YOLO v3 (three layers) and YOLO v3 (five layers) have different prediction layers, both of them adopt Darknet-53 as the basic backbone. The second goal is to evaluate the effect of training image sizes on YOLO v4 models. The default training image size of YOLO v4 is 416 × 416. The authors of YOLO v4 suggest increasing the image size with the multiple of 32, and a suitable, larger training image size should be 608 × 608 to recognize more details by the model [35]. The third goal is to evaluate the performance of different sizes of YOLO v5 models. In theory, the larger size model usually has a higher accuracy than smaller ones, while its speed is slower. Therefore, it is meaningful to test all size models of YOLO v5 and present an overall picture of their performance.

It should be noted that, for the first three goals, only one version of YOLO was used to test one goal. More specifically, only YOLO v3 models were tested for layers, YOLO v4 models for the training image size, and YOLO v5 models for the model size, as listed in Table 1 from Experiment No. 1 to 8. The last goal is to test YOLO models on blurring face images based on YOLO v5x. For protecting personal information, many videos and images may hide the face information; therefore, the performance of the model on blurring face images needs to be tested.

#### 2.4.2. Blurring Face Test

Face information belongs to personal private data. Protecting the face information becomes more necessary. For avoiding leaking any sensitive facial information, some videos captured by surveillance cameras would be blurred by algorithms before any further processing. Therefore, the performance of YOLO models on blurring faces is also valid to be evaluated.

Face detection has been widely researched over the past two decades. In the early stage of face detection, the algorithms were based on handcrafted features such as Haar-like features [36], the histogram of oriented gradients (HOG) [37]. Such algorithms’ performance is limited by the expression ability of the features, which results in the failure of detection. Besides, deep learning methods have been introduced to face detection in recent years. Two-stage detectors, like Face R-CNN [38], shows high accuracy, while its long processing time is the main limitation. On the contrary, the advantages of YOLO based single-stage detector are the ability of real-time processing and detecting small instances, especially for face detection. In this paper, YOLO-face [39] is employed to detect the face. Then the Gaussian Blurring is used to create a blurring kernel. The Gaussian kernel size can be denoted as *k*, and the two-dimensional kernel can be defined as in Equation (3).

(3) $$G\left(x,y,\sigma_{1},\sigma_{2}\right)=\frac{1}{2\pi \sigma_{1}\sigma_{2}}e^{-\frac{x^{2}+y^{2}}{2\sigma_{1}\sigma_{2}}}$$

where $x,y\in [\frac{k-1}{2},\frac{k+1}{2}]$, $\sigma_{1},\sigma_{2}$ are the standard deviation in the *x* and *y* directions. This kernel is further used as a convolution matrix, which is applied to the original image.

#### 2.4.3. Training Process

The whole training work was implemented on the cloud training platform, Didi Yun, with a 24 GB Tesla P40 graphics card. After the training, both local PC CPU and cloud-based GPU platforms were used to test the performance of different deployment methods. The CHV dataset was split into three portions for training, validation, and testing based on the percentage of 80%, 10%, and 10% respectively.

The training of YOLO v3/4 is based on the darknet environment developed by AlexeyAB et al. [35]. The training process contains three steps. Step 1: select the desired model and set the configure file according to the targeted objects; Step 2: adopt the pre-trained weights that provide initial parameters inside the networks and accelerate the training process; Step 3: set the training parameters and start the training process. The stochastic gradient descent (SGD) was adopted with an initial learning rate of 0.001. The max batch was calculated from classes × 2000 [4] (12,000 in this study). The batch with the highest mAP was recorded as the best weight. Other hyperparameters are listed in Table 2. Additionally, YOLO v5 was trained in a PyTorch environment constructed by Ultralytics [29]. The training process was similar to YOLO v3/4 and the hyperparameters are shown in Table 2. The platform information is listed in Table 3.

### 2.5. Metrics for Performance Evaluation

**Figure 7:**
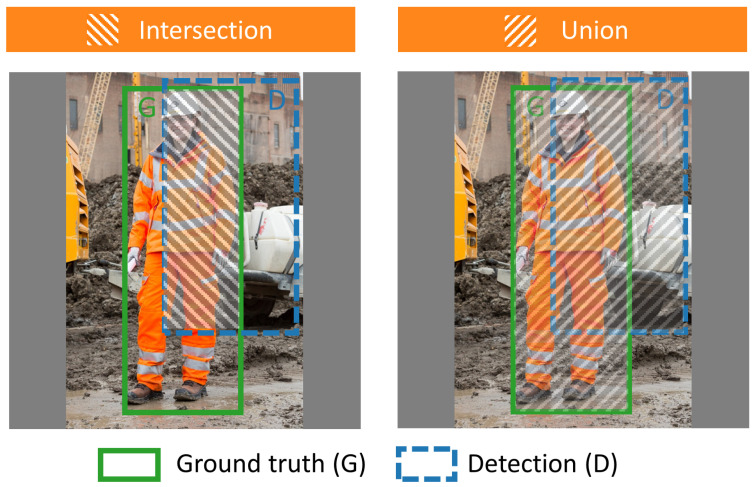
Examples of intersection and union.

A basic metric to measure the performance of object detection algorithms is intersection over union (IoU), as shown in Figure 7. *IoU* represents the percentage of overlap between two boxes, the ground truth box (*G*) and the detection box (D). It is calculated using Equation (4) [40].

(4) $$IoU=\frac{Intersection}{Union}=\frac{G\cap P}{G\cup P}$$

After obtaining an IoU, the confusion matrix criteria, e.g., True Positive (TP), False Positive (FP), True Negative (TN) and False Negative (FN), are then calculated to quantify the accuracy. For TP, the class of ground truth should be the same as the class of detection, and its IoU should be larger than 50%. TP refers to the corrected detection to the class. On the contrary, if the detection owns the same class as the ground truth and its IoU is smaller than 50%, it is regarded as FP. This means an uncorrected detection case. Meanwhile, if there is a ground truth box, while the model does not make any detection. This situation is categorized as FN, which means that the instance cannot be detected. Finally, for most situations, the background does not have any ground truth, as well as detection, which is classified as TN.

*Precision* represents the ability of a model to identify only the relative objects [40]. It is the percentage of correct positive predictions among all detections, as shown in Equation (5).

(5) $$Precision=\frac{TP}{TP+FP}=\frac{TP}{all\;detections}$$

On the other hand, *Recall* represents a model’s ability to find all the relevant cases, and the percentage of true positives among all ground truths, as shown in Equation (6).

(6) $$Recall=\frac{TP}{TP+FN}=\frac{TP}{all\;ground\;truths}$$

The Precision × Recall curve is another way to evaluate the performance of an object detector, as the confidence is changed by plotting a curve for each class. The Precision × Recall curve is plotted by calculating the precision and recall values of the accumulated TP or FP [40]. All detections are sorted in descending order of their corresponding confidence level. Then, starting from the detection with the highest confidence level, the precision and recall are calculated and plotted [41].

The average precision (AP) is the area under the Precision × Recall curve for each class. This can be calculated using Equation (7) [42]. After this, the mean average precision (mAP), presenting the overall performance of the model in the detection of possible classes, could be estimated by calculating the mean of all classes’ AP.

(7) $$AP=\sum_{i=1}^{n}p\left(i\right)\Delta r\left(i\right)$$

where *n* is the total number of detections, *i* is the rank of a particular detection in the list of sorted detections, p(i) is the precision of the sub-list ranged from the first to *i*th detection, and Δr(i) is the change in recall from (*i*− 1)th to *i*th detection.

Two platforms are constructed for a training and speed test. One is a local personal computer with CPUs, which is not powerful enough for training work and can only be used to test the speed of models on personal equipment. Another one is a cloud high-performance server with a 24 GB Tesla P40 graphics card, which is constructed for training and testing the speed on high-performance equipment. The parameters of two platforms are listed in Table 3.

The speed of this study refers to the time consumed by processing an image by the model. More specifically, the time does not contain the time taken to read and write an image. Additionally, the final speed is calculated based on the average of 133 test images.

## 3. Results and Discussions

### 3.1. Results

**Figure 8:**
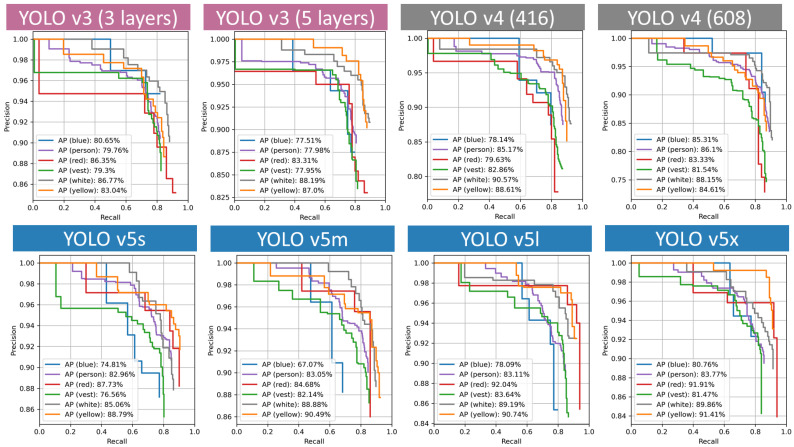
Precision × Recall curves.

For a Precision × Recall curve, the prediction ability is better when the precision maintains a large value with the increase in the recall. Therefore, the curve presenting a better performance is closer to the right corner. The Precision × Recall curves are presented in Figure 8. The AP of four classes (blue, person, red, vest) on the YOLO v3 (3 layers) model is higher than that on YOLO v3 (5 layers) model. Similarly, the AP of vest, white, yellow of YOLO v4 (416) is greater than that of YOLO v4 (608). A larger size YOLO v5 model has a better performance. The smallest one’s AP ranges from 70% to 80%. However, for the YOLO v5x model, two classes’ APs are over 90% and all AP is above 80%, demonstrating a more accurate and balanced result.

**Figure 9:**
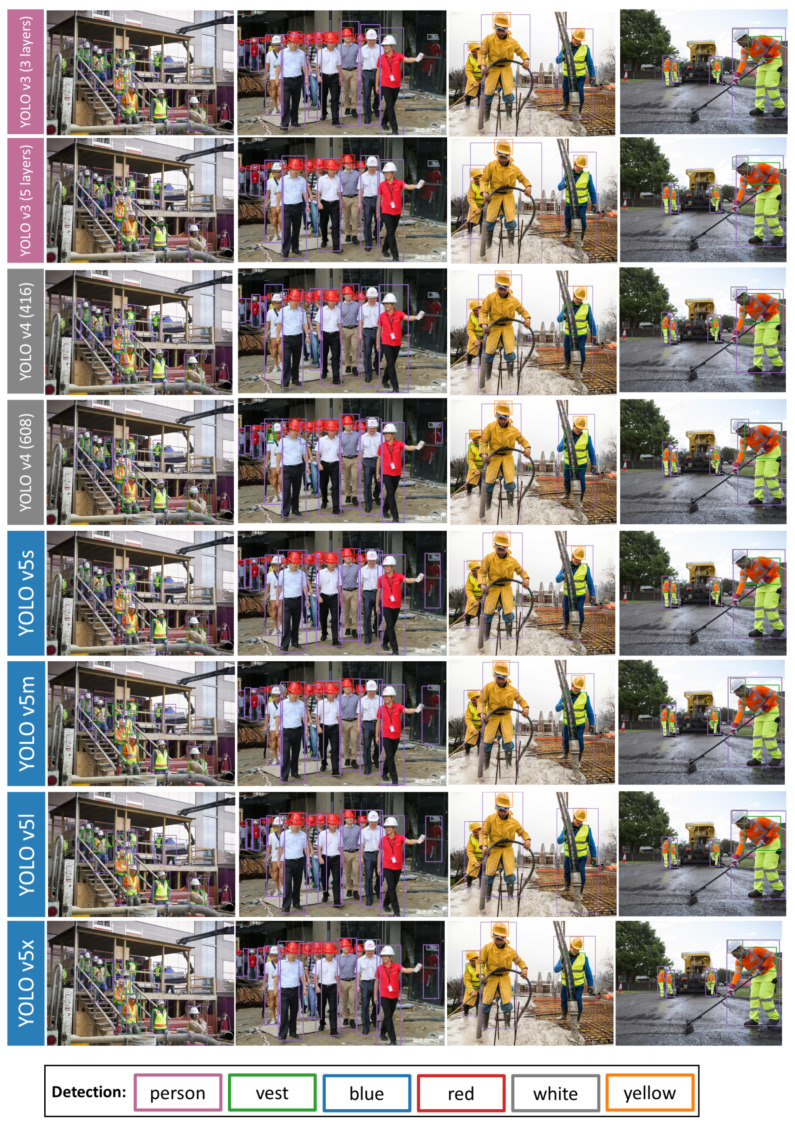
Examples of detection results.

Meanwhile, examples of detection results for different models are presented in Figure 9. The left column is very challenging, as it contains crowded people with different working distances to cameras. Detecting close-range objects and far-away objects at the same time is difficult for many detectors [43,44]. YOLO models perform well by detecting most objects correctly in this image.

The middle-left image is an example that can be used to explain the ability of each model. Firstly, some people standing further back are blocked by people in the front. From the results, YOLO v3 models can only make predictions for large-size instances, while many objects, especially for the helmet colors, are missed by YOLO v3 models. On contrary, YOLO v5 models are relatively powerful, which can detect all instances and even the objects in the right black mirror.

The right two images contain various gestures and angles. Workers may bend over, raise legs and bow heads. They also stand at random angles. Meanwhile, occlusions happen in all images, where workers are blocked by people standing in front of them. Only two models, YOLO v4 (416) and YOLO v5x, detect them correctly.

### 3.2. Different Layers

Two YOLO v3 models with varied layers are tested. In theory, the model with more layers has a stronger ability to detect small objects. However, the results show that the YOLO v3 (3 layers)’s mAP is only slightly higher than YOLO v3 (5 layers)’s. Four classes of AP (person, vest, blue, red) in YOLO v3 (three layers) is also larger than the five-layer classes. Additionally, due to the extra two layers, the five-layers’ weight is slightly higher, as shown in Table 4. Additionally, the model with more layers has a longer processing time, because the structure is larger and more complicated after adding extra layers. In experiments, YOLO v3 (three layers) needs 27.25 ms to process an image on GPU, which is 27% faster than YOLO v3 (five layers). The results are consistent with the theoretical analysis.

### 3.3. Different Train Size

The default training image size is **416 × 416**. In theory, more details can be learned during the training process when feeding large-size images. In this experiment, the training image size is increased by 42% to **608 × 608**. The results are listed in Table 5. It is observed that there is little difference after changing the image size. They almost have the same mAP and processing time.

### 3.4. Different Model Sizes

YOLO v5 models provide four sizes, e.g., extra-large (v5x), large (v5l), middle (v5m), small (v5s). All four YOLO v5 models maintian the same network structure. However, they have different model depth and model width, which are jointly used for model scaling and compound scaling and result in different model sizes. YOLO v5x has the largest depth and width, with the biggest weight files, 178 MB. YOLO v5s multiples a smaller model depth and width, whose weight is only 15 MB, only 8% of the largest one. Their weight sizes and the AP for each class are presented in Table 6. Four classes in YOLO v5x, including person, blue, white and yellow, have the highest AP. On the contrary, YOLO v5s, the smallest model, has the lowest overall AP.

**Figure 10:**
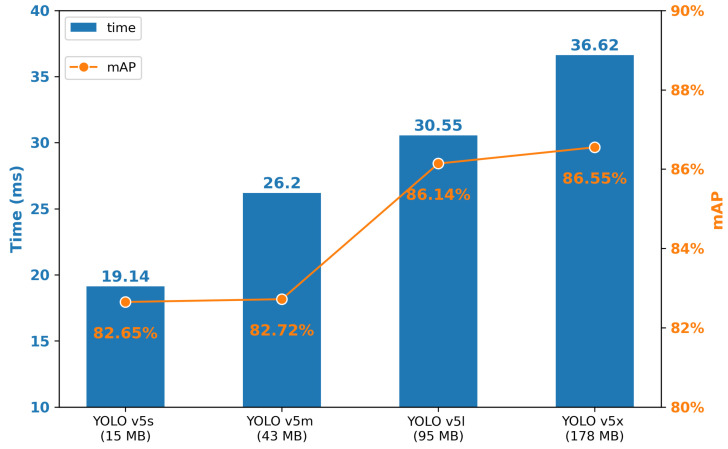
Process time (GPU) and mAP of each YOLO v5 models.

Figure 10 presents the mAP and speed of YOLO v5 series models. The mAP and processing time rises with the increase in weight. Specifically, for mAP, YOLO v5x has the greatest results, 86.55%, 3.9% higher than the lowest one. On the other hand, to process an image on GPU, YOLO v5s has the shortest time, 19.14 ms, which is almost half of the YOLO v5x’s. In another calculation, the FPS of YOLO v5s is 52, which meets the real-time processing requirement.

In fact, YOLO v5x (86.55%) and YOLO v5l (86.14%) almost have the same mAP, while the YOLO v5l’s speed is faster. The same situation occurs between YOLO v5s and YOLO v5m. It is more practical to choose the faster one, which has a similar correctness.

### 3.5. Where Are Wrong Detections?

**Figure 11:**
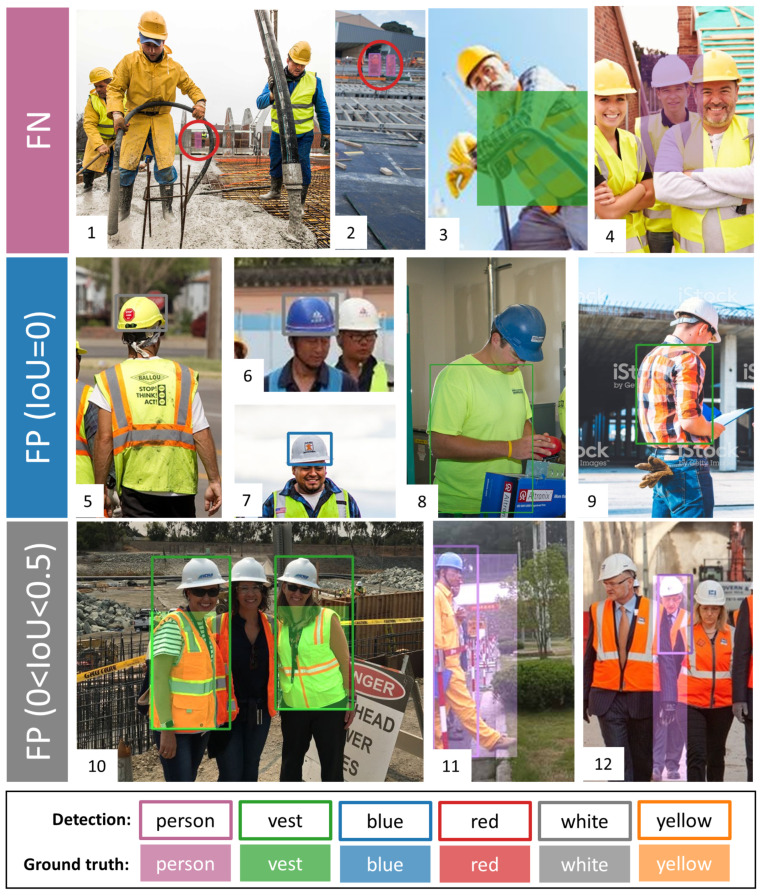
Wrong detections in YOLO v5x.

To explore the model’s performance in more depth, it is necessary to see their incorrect detections, which could provide future directions for improvement. YOLO v5x, the best correctness one, is selected to analyze the incorrect detections. The confusion matrix of YOLO v5x is presented in Table 7. Unlike the traditional confusion matrix, FP is categorized into two parts based on the IoU value. When IoU = 0, the FP prediction is far away from the ground truth. Similarly, when IoU is smaller than 0.5 but bigger than 0, the overlap between the prediction and the ground truth is not enough to be regarded as TP. Some incorrect detections in YOLO v5x are selected as the samples used to present the typical errors, as shown in Figure 11.

FN happens when the ground truth is not detected by the model. As is shown in Table 7, YOLO v5x misses 65 person instances, 35 vest instances and 38 helmet color instances. From the first two images in Figure 11, the miss instances are too small to be detected in the background (red-circle instances). Instead of missing all instances, the problem is that the model cannot entirely detect the small instances. Furthermore, FN also occurs when people have strange gestures (the third one) and the instance is partial occlusion (the fourth one).

Additionally, the second row in Figure 11 shows the detection errors with FP (IoU = 0). Firstly, there are 30 wrong-color detections, as shown in the fifth, sixth and seventh images. Meanwhile, the model also mis-detects the normal green and orange shirts as a vest (the eighth and ninth images). Indeed, the vest color is usually green and orange, which may cause the model to detect the normal green and orange clothes as vests.

Lastly, the third row presents the error of FP (0 < IoU < 0.5). Visually, the detection is placed in the right position, while the size is not appropriate. For the vest class, the detection box is usually much larger than the ground truth. For the person class, the detection does not contain the whole annotated body. Indeed, there are 63 instances of FP (0 < IoU < 0.5), which indicates that the model is sensitive to the IoU threshold. In fact, there are 35 instances where the IoU is between 0.4 and 0.5. After adjusting the IoU from 0.5 to 0.4, the number of FP decreases 28 and the mAP increases by 3%, to 89.16%.

### 3.6. All YOLO Models

The previous sections discuss the performance of each version’s models. In this section, a comparison of all YOLO models will be discussed. Their confusion matrix is listed in Table 8, where the blue presents the largest TP for each class, and the red refers to the smallest FP and FN. The person class owns the largest number of instances, while the number of red and blue instances is comparatively small.

**Figure 12:**
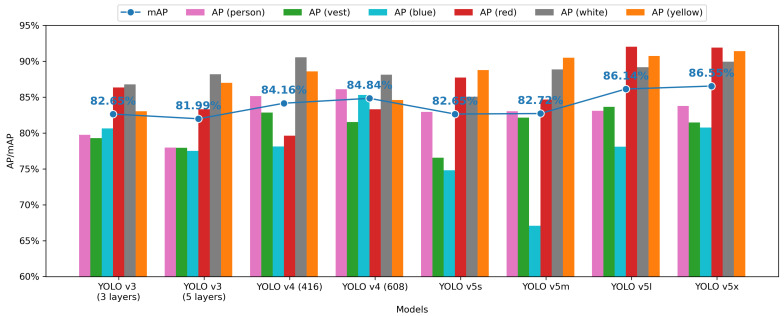
Mean average precision in each model.

The AP for each class and the mAP for each model are presented in Figure 12. YOLO v5x obtains the best mAP, 86.55%, while YOLO v3 (five layers) performs relatively poorly with an mAP of 81.99%. For each version’s models, the mAP of YOLO v3 models is around 82%, and YOLO v4 models’ mAP improved by 2% compared with YOLO v3 models. The mAP of YOLO v5 models ranges from 82% to the highest mAP of 86.55%. Meanwhile, the YOLO v3 model has a balanced performance in the detection all classes, while the YOLO v5s model is relatively poor at detecting blue helmets, which affects its overall performance.

**Figure 13:**
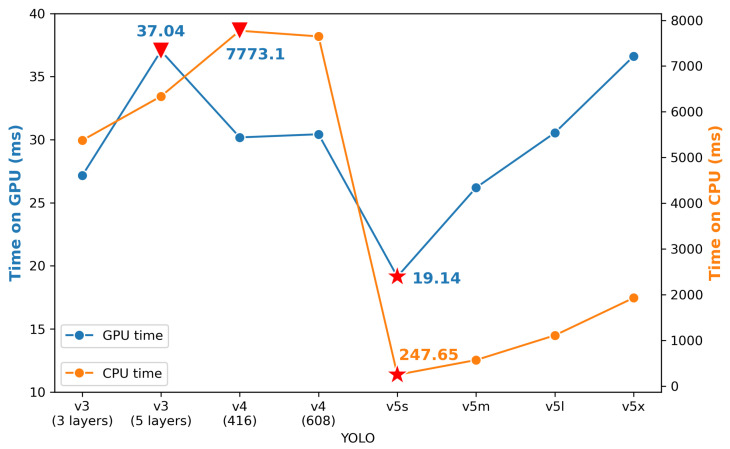
Average time for processing one image in each model.

On the other hand, the speed results on both GPU and CPU are presented in Figure 13. YOLO v5s takes less time than other models on both GPU and CPU, while YOLO v3 (five layers) and YOLO v4 (416) need the longest time for GPU and CPU, respectively. Meanwhile, processing GPU is about ten times faster than processing CPU. Even the slowest GPU speed, 37.04 ms/image, would meet the real-time detection requirement. However, for CPU, it can, at most, deal with four images per second, which is far away from the real-time detection. This comparison would contribute to the reference used for selecting models for the practical situation.

### 3.7. Blurring Face Test

**Figure 14:**
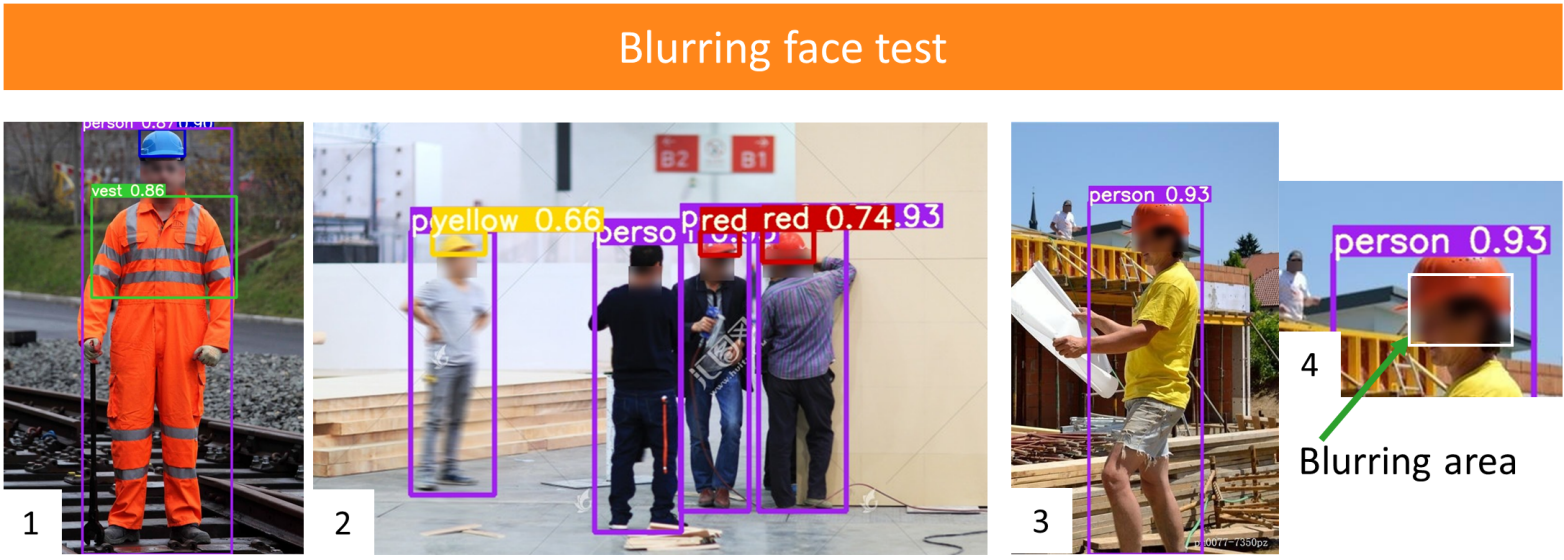
Blurring face test on YOLO v5x.

YOLO v5x is adopted to test the blurring face images. It should be emphasized that YOLO v5x is trained on clear-face images. The test samples are presented in Figure 14. During the prediction process, the model works well under most situations, as in the 1st and 2nd images. All the desired classes are detected, and the results are not affected by the blurring areas. However, if the algorithm blurs the helmet area, PPE detection may fail to detect the helmet class, as shown in the 3rd image (the original clear instance could be detected by YOLO v5x). The blurring mask covers almost half of the helmet area, and the model did not recognize the appearance of the helmet.

Furthermore, the statistic results between clear face images and blurring face ones are presented in Table 9. As expected, the AP of person and vest classes is almost the same, while the blurring AP of the other four helmet classes is lower than the clear ones. Meanwhile, the mAP of blurring images also decreases by 7%. Therefore, it can be concluded that the blurring face does not affect the performance of the person and vest classes. However, it does decrease the accuracy of helmet colors. An accurate face detection is required for face blurring.

### 3.8. Comparison with the State-of-the-Art

The section reports the results to compare the proposed models with other state-of-the-art deep-learning methods, based on the same datasets. We collected two public helmet datasets, including Pictor-v3 (crowd-sourced part) [42] and GDUT-HWD [22], for performance comparison. After selecting test images from each dataset, we manually relabelled test images because the previous labelled categories are not matched with this study (this study detects more classes).

The test images were selected from the previous studies or randomly selected according to the dataset situation. Pictor-v3 consists of two parts of the images, named crowd-sourced and web-mined ones, while only the crowd-sourced part was released. This part was collected from real construction sites, where the workers are far away from cameras and make various gestures. We adopt these 152 test images in [42] for testing. Most images in GDUT-HWD were collected from the internet, where some images are not from construction sites and people usually stand closer to cameras compared with Pictor-v3 (crowd-sourced part). We randomly selected 300 images from GDUT-HWD as another test dataset. Then, the test images were fed into the best-performing model in this study, YOLO v5x. The results are listed in Table 10.

Generally, from Table 10, YOLO v5x has a higher AP in each class and higher mAP in each dataset, compared to the previous research, for both datasets. More specifically, YOLO v5x achieves 81.79% mAP in Pictor-v3 (crowd-source part), which increases by nearly 10% mAP compared with YOLO v3 [42]. Additionally, YOLO v5x improves the AP of each helmet color in GDUT-HWD (>80%), and the mAP is also increased by 11% compared with Pelee-RPA [22]. It should be noted that Pelee-RPA cannot detect person and vest.

## 4. Conclusions

This paper introduced, trained, and evaluated a number of effective PPE detectors based on the newest YOLO models. The main contribution of this study, associated with the corresponding key findings, is listed as follows:

1. A new dataset, CHV dataset, was constructed to detect the desired six classes of PPE. It contained 1330 high-quality images from the real construction sites, selected from more than 10,000 images, based on strict criteria. To foster future innovative research work, the dataset was publicly available on the GitHub page at https://github.com/ZijianWang1995/ppe_detection (accessed on 13 May 2021);
2. This paper expanded the detection scope to more PPE characteristics, including people, four helmet colors and the vest. The detection results could measure the safety compliance, and contribute to the management;
3. Different versions of YOLO with different parameters were tested systematically in terms of accuracy and speed. For YOLO v3 models, different detection layers were tested, while an increased number of layers could not improve performance. For YOLO v4 models, the increase in training image size could not contribute to a better performance. Additionally, YOLO v5x owned the best mAP (86.55%), and YOLO v5s had a faster processing speed for one single image (52 FPS). Even compared with previous studies, YOLO v5 models still had the best performance;
4. Detection errors for YOLO v5x were analyzed in more depth. In the False Negative (miss detection) cases, it was found that small, blocked, and strange-gesture instances were hard to detect. In the False Positive (wrong detection) cases, on the one hand, the detector mis-classified the helmet colors or mistakenly regarded green clothes as vests. On the other hand, while the detection class was correct, the detection size was not appropriate for the ground truth size;
5. YOLO v5x was adopted to test blurring face images. The detection of the vest and person was not affected by the blurring areas, while the average precision of four helmet colors decreased by around 7% when the blurring area covered the helmet part.

However, there are several limitations to the current work, which could provide future improvement directions. One limitation is that the model sometimes predicted the helmet colors incorrectly and detected the regular green T-shirt as the vest. Expanding the dataset with more t-shirt and helmet images could reduce the errors. Another limitation of YOLO models is that it is hard to detect all small instances from a relatively long distance, especially when there are some large objects within a close range. Small object detection was a bottleneck for all current deep learning detectors, where adjusting or creating architecture is a potential way of solving this problem. Lastly, although the model was expanded to six different PPE classes, it was also meaningful to detect more necessary PPE in the future, like masks, glasses and gloves.

## Figures and Tables

**Table 1:** Experimental goals (one YOLO version for one goal).

No.	Goal	Model	Note
1	Layer	YOLO v3 (3 layers)	Default configure
2		YOLO v3 (5 layers)	Two more layers
3	Training image size	YOLO v4 (416)	Default configure
4		YOLO v4 (608)	Larger training image size
5	Model size	YOLO v5s	Small
6		YOLO v5m	Medium
7		YOLO v5l	Large
8		YOLO v5x	Extra large
9	Blurring face	YOLO v5x	Protect private information

**Table 2:** Training hyperparameters.

Model	DL Library	Optimizer	Initial Learning Rate	Momentum	Decay	Batch Size
YOLO v3/4	Darknet	SGD	0.001	0.949	0.0005	64
YOLO v5	PyTorch	SGD	0.01	0.937	0.0005	32

**Table 3:** Platform parameters.

Platform	Location	OS	GPU	GPU Size	CPU Cores	RAM
Traning	Cloud	Ubuntu 16.04	Tesla P40	24 GB	8	48 GB
Test	Local	macOS Catalina	None	None	4	8 GB

**Table 4:** Performance of YOLO v3 models.

Model	Weight Size	AP						mAP	Time (ms)	
		Person	Vest	Blue	Red	White	Yellow		GPU	CPU
YOLO v3 (3layers)	246 MB	79.76%	79.30%	80.65%	86.35%	86.77%	83.04%	82.65%	27.15	5375.02
YOLO v3 (5layers)	248 MB	77.98%	77.95%	77.51%	83.31%	88.19%	87.00%	81.99%	37.04	6338.74

**Table 5:** Performance of YOLO v4 models.

Model	Train Image Size	AP						mAP	Time (ms)	
		Person	Vest	Blue	Red	White	Yellow		GPU	CPU
YOLO v4 (416)	416 × 416	85.17%	82.86%	78.14%	79.63%	90.57%	88.61%	84.16%	30.18	7773.10
YOLO v4 (608)	608 × 608	86.10%	81.54%	85.31%	83.33%	88.15%	84.61%	84.84%	30.43	7648.62

**Table 6:** Correctness and weight size of YOLO v5 models.

Model	Weight Size	AP					
		Person	Vest	Blue	Red	White	Yellow
YOLO v5x	178 MB	83.77%	81.47%	80.76%	91.91%	89.96%	91.41%
YOLO v5l	95 MB	83.11%	83.64%	78.09%	92.04%	89.19%	90.74%
YOLO v5m	43 MB	83.05%	82.14%	67.07%	84.68%	88.88%	90.49%
YOLO v5s	15 MB	82.96%	76.56%	74.81%	87.73%	85.06%	88.79%

**Table 7:** YOLO v5x confusion matrix.

Category		Person	Vest	Blue	Red	White	Yellow
TP		385	182	36	47	161	135
FN		65	35	8	3	15	12
FP	IoU = 0	13	15	1	6	16	7
	0 < IoU < 0.5	32	19	2	3	4	3

**Table 8:** Confusion matrix of YOLO models.

Models	Person			Vest			Blue			Red			White			Yellow		
	TP	FP	FN	TP	FP	FN	TP	FP	FN	TP	FP	FN	TP	FP	FN	TP	FP	FN
YOLO v3 (3 layers)	369	39	81	179	26	38	36	2	8	46	8	4	155	17	21	125	16	22
YOLO v3 (5 layers)	363	47	87	177	35	40	35	5	9	44	9	6	158	16	18	129	14	18
YOLO v4 (416)	393	56	57	189	44	28	35	5	9	42	12	8	163	23	13	132	23	15
YOLO v4 (608)	401	79	49	189	64	28	38	5	6	43	16	7	160	35	16	128	25	19
YOLO v5s	382	39	68	174	30	43	34	5	10	45	6	5	152	21	24	133	12	14
YOLO v5m	381	42	69	185	27	32	30	4	14	43	7	7	158	20	18	136	19	11
YOLO v5l	382	48	68	188	24	29	35	6	9	47	8	3	160	13	16	135	11	12
YOLO v5x	385	45	65	182	34	35	36	3	8	47	9	3	161	20	15	135	10	12

**Table 9:** Correctness of blurring face test on YOLO v5x.

Face	Models	Person	Vest	Blue	Red	White	Yellow	mAP
Clear	YOLO v5x	83.77%	81.47%	80.76%	91.91%	89.96%	91.41%	86.55%
Blurring	YOLO v5x	82.78%	82.46%	73.78%	74.59%	78.46%	85.17%	79.55%

**Table 10:** Comparison among YOLO v5x and other deep learning methods based on the same datasets.

Dataset		Pictor-v3 (Crowd-Sourced Part)		GDUT-HWD	
**Method**		**YOLO v3 [42]**	YOLO v5x *	**Pelee-RPA [22]**	YOLO v5x *
Class	Person	×	85.74%	×	86.71%
	Vest	84.96%	×	×	81.58%
	Blue	×	×	77.35%	82.74%
	Red	×	87.39%	68.58%	81.87%
	White	×	72.98%	74.64%	86.56%
	Yellow	×	81.05%	78.48%	81.10%
	Helmet	79.81%	80.47%	72.34%	83.07%
mAP		72.30%	81.79%	72.34%	83.43%

Symbol: * From this study. Not available.

## Data Availability

CHV dataset, presented in this study, is available at https://github.com/ZijianWang1995/ppe_detection (accessed on 13 May 2021). All images in CHV dataset are from two open datasets, GDUT-HWD [22] at https://github.com/wujixiu/helmet-detection/tree/master/hardhat-wearing-detection (accessed on 13 May 2021) and SHWD [33] at https://github.com/njvisionpower/Safety-Helmet-Wearing-Dataset (accessed on 13 May 2021).
